# A Soft Tactile Sensor Based on Magnetics and Hybrid Flexible-Rigid Electronics

**DOI:** 10.3390/s21155098

**Published:** 2021-07-28

**Authors:** Miguel Neto, Pedro Ribeiro, Ricardo Nunes, Lorenzo Jamone, Alexandre Bernardino, Susana Cardoso

**Affiliations:** 1 Instituto de Engenharia de Sistemas e Computadores—Microsistemas e Nanotecnologias, 1000-019 Lisbon, Portugal; pribeiro@inesc-mn.pt (P.R.); scardoso@inesc-mn.pt (S.C.); 2 Instituto Superior Técnico, Universidade de Lisboa, 1049-001 Lisbon, Portugal; rnunes@isr.tecnico.ulisboa.pt (R.N.); alex@isr.tecnico.ulisboa.pt (A.B.); 3 Advanced Robotics at Queen Mary, School of Electronic Engineering and Computer Science, Queen Mary University of London, London E1 4NS, UK; l.jamone@qmul.ac.uk; 4 Institute for Systems and Robotics (ISR), 1049-001 Lisbon, Portugal

**Keywords:** flexible hybrid electronics, magnetic tactile sensor

## Abstract

Tactile sensing is crucial for robots to manipulate objects successfully. However, integrating tactile sensors into robotic hands is still challenging, mainly due to the need to cover small multi-curved surfaces with several components that must be miniaturized. In this paper, we report the design of a novel magnetic-based tactile sensor to be integrated into the robotic hand of the humanoid robot Vizzy. We designed and fabricated a flexible 4 × 2 matrix of Si chips of magnetoresistive spin valve sensors that, coupled with a single small magnet, can measure contact forces from 0.1 to 5 N on multiple locations over the surface of a robotic fingertip; this design is innovative with respect to previous works in the literature, and it is made possible by careful engineering and miniaturization of the custom-made electronic components that we employ. In addition, we characterize the behavior of the sensor through a COMSOL simulation, which can be used to generate optimized designs for sensors with different geometries.

## 1. Introduction

Object manipulation is an essential skill for robots to interact with the external world, especially in environments designed for human beings [1]. It is not a trivial task because it requires the successful coordination of body parts (i.e., head and eyes, arm, hand, fingers) and sensory channels (i.e., vision, touch, proprioception).

One crucial component in object manipulation, but still not fully developed in robotic manipulation, is tactile sensing [1,2]. In fact, although tactile sensing has been an active research area for more than three decades [3], the effective integration of tactile sensing systems into robots is still limited [4], even for robotic hands [5]. One of the biggest challenges is to cover small multi-curved surfaces, such as the fingertip of a robot hand, with several sensors so that the contact forces can be measured on multiple locations simultaneously. This challenge can be tackled by using miniaturized components and flexible electronics, in addition to clever design solutions that would minimize the number of components without sacrificing the quality of the measurements. In the rest of the manuscript, we report the design and realization of a physical sensor that has been successfully integrated in the finger of a humanoid robot (i.e., Vizzy [6]), in addition to the simulations that characterize the fabricated tactile sensor. Our main contributions are: a novel design for magnetic-based tactile sensors and a physical realization of a miniaturized device.

The rest of the paper is organized as follows: In Section 2 we discuss the most relevant related works, and we outline our main contributions. In Section 3 we describe the materials and methods used in our research. In Section 4 we report on an extensive set of experiments aimed at characterizing the behavior of the sensor, both in simulation and with a real-world prototype. In Section 5 we conclude the paper by summarizing our main findings and discussing the most promising extensions to this work.

## 2. Related Work and Our Contribution to the State of the Art

### 2.1. Transduction Methods and Sensor Design

Tactile sensing requires transforming a mechanical deformation into an electronic signal. To achieve this, several transduction methods have been proposed [7]: e.g., capacitive [8], piezoresistive [9], piezoelectric [10], optical [11,12,13], and magnetic [14,15]. The magnetic tactile sensors usually benefit from high robustness, low or inexistent mechanical hysteresis, low cost, and are relatively easy to assemble [16]. 

Ideally, it would be desirable for a sensor to show: high sensitivity, high signal-to-noise ratio, low hysteresis, and high spatial and temporal resolution [17]. All these characteristics must be maintained when the sensor is mechanically and electronically integrated within the target robotic system.

Each transduction method has some benefits and some drawbacks, and the best choice often depends on the application. For example: capacitive sensors often show a low signal-to-noise ratio, piezoresistive sensors suffer from hysteresis, piezoelectric sensors offer low spatial resolution, and optical sensors are difficult to miniaturize [7]. With respect to other solutions, magnetic tactile sensors can show very high sensitivity [14,18], that might come at the cost of a smaller range of force measurements [19]; in addition, they benefit from high signal-to-noise ratio, and low mechanical hysteresis [20]. Notably, low-cost versions of such sensors, which are also easy to assemble, can be produced as well [16].

Two main working principles for magnetic tactile sensors have been proposed in the literature: magnetic field [21] and electromagnetic induction [22]. The most common is the magnetic field approach, mainly because the sensors of that kind usually have lower power consumption and are not susceptible to stray capacitance issues as opposed to those based on electromagnetic induction. 

**Figure 1:**
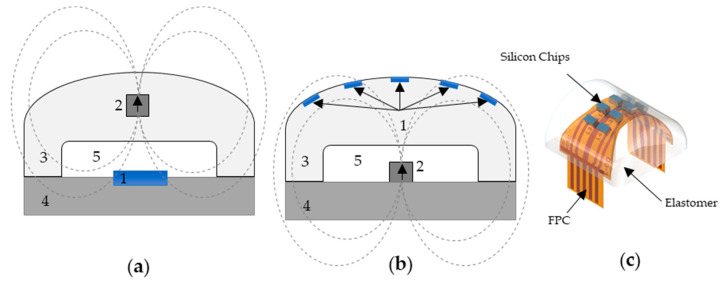
Schematic view of the devices’ working principle: (**a**) concept currently integrated in Vizzy hand [16]; (**b**) the flexible sensing matrix concept. The numbers in the figure stand for: 1—sensors (blue); 2—cylindrical Nd permanent magnet; 3—polymeric silicone part; 4—robotic finger; 5—air gap; (**- - -**)—magnetic field lines for the permanent magnet. (**c**) 3D view of the FPC with the Si chips embedded in the elastomer part.

The magnetic field approach requires a magnetic sensor fixed onto the robot finger and a permanent magnet (PM) embedded into an elastomeric part [14] (see Figure 1a). Applying a force on the elastomeric part changes the relative orientation between the sensor and the permanent magnet, resulting in a change in the sensor output. However, this approach assumes knowledge of the point and area of contact (which are often not known in robotic applications outside structured environments), or that the area of contact is larger than the whole surface of the sensor; instead, we would like a sensor that could automatically measure the contact area and location, in addition to the contact force, as these are essential requirements for grasping activities [23]. To overcome this limitation, we propose a solution where a cylindrical neodymium permanent magnet (1 × 1 mm) (2) is fixed on the robot finger (4) while a flexible sensor matrix (FSM) ((1) on Figure 1b) is positioned at the surface of the silicone (3). To the best of our knowledge, this design is novel, and it thus makes an original contribution to the state of the art.

### 2.2. Device Manufacturing and Miniaturization

Integration of magnetoelectronic devices in flexible substrates [24,25] does not match the performance of spintronic devices in rigid substrates, and these approaches include unreproducible processes not compatible with small dimension devices [26]. The impact of film strain, bending, and thermal stress on the sensor transport curves have shown to have significant contributions for films deposited on Si [27], polyimide [28], or stretchable substrates [24]. To reduce the complexity introduced by mechanical stresses on these devices, we use a hybrid flexible-rigid approach, where the sensors are processed in silicon wafers and connected using a flexible printed circuit (FPC) board. 

In our magnetic tactile sensor, eight Si rigid chips are arranged in a 4 × 2 matrix covering the surface area of the elastomeric part. Each Si chip comprises six spin-valve sensors connected in series and two contact pads to connect to the FPC (Figure 1b,c). Because the Si chips are rigid, the surface is assumed to be locally flat, enabling simplified models to evaluate the tactile sensor performance during characterization and simulations. Overall, the sensor has a total of 8 chips within a surface area of 1.77 cm^2^, which is equal to a spatial resolution of about 4.5 taxels/cm^2^, which is state of the art for this kind of sensor: therefore, we consider the manufacturing details of our electronic device as an important contribution of this work.

## 3. Materials and Methods

### 3.1. Device Manufacturing

**Figure 2:**
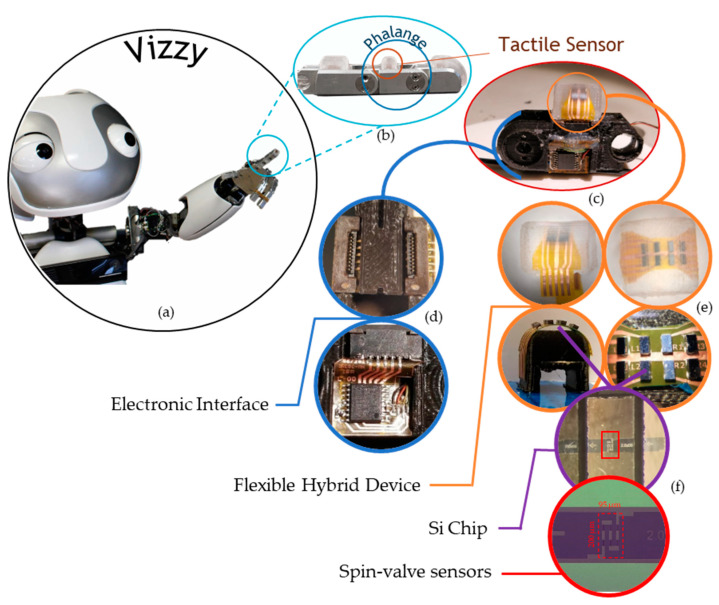
The robot, the finger, the sensor, the sensing elements, and their integration. (**a**) Vizzy; (**b**) a finger from Vizzy with three phalanges, each with a tactile sensor; (**c**) redesigned middle phalange part to integrate (**d**) the electronic interface and (**e**) flexible hybrid device with the flexible sensing matrix and the (**f**) Si chips with the spin-valve sensors.

The tactile sensor’s design is compatible with the Vizzy humanoid robot hands (Figure 2a) to be located at one of the phalanges, together with reliable electronic components (Figure 2b,c). This work describes the fabrication of several parts required for the tactile solution’s success: a rigid chip with microfabricated magnetic sensors, a flexible cable for contact interconnection, and an elastomer cap layer embedding the sensors. The permanent magnet is attached to the finger’s rigid frame, therefore not moving during the tactile actuation.

The sensing matrix consists of a matrix of 4 × 2 rigid silicon chips (Figure 2d), each containing six microfabricated magnetoresistive spin valve sensors [29] (Figure 2f), in a flexible printed circuit board (FPC) and embedded in a silicone cap, all fabricated at INESC-MN.

Thanks to the miniaturization of our spin valve sensors, we can fit eight chips in this design; this creates a tactile sensor area that has a density of sensitive elements (i.e., taxels) larger than any other magnetic tactile sensor to date. The state-of-the-art solution for magnetic flexible skin, uSkin [30], has 2.3 sensors/cm^2^ (16 sensors/2.7 × 2.6 cm^2^) [30], while our proposed solution has 4.5 sensors/cm^2^ (8 sensors/1.77 cm^2^), which is more than double. 

After fabrication, we followed up with electronics and integration, testing and finite element simulation. We identify practical challenges regarding each phase and provide feedback on future iterations as well as for the development of new tactile sensors.

### 3.2. Rigid Chips with the Magnetoresistive Sensors

**Figure 3:**
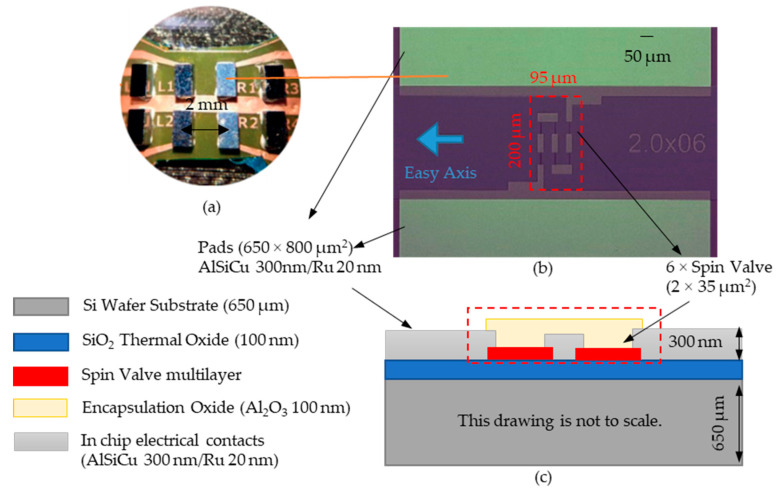
(**a**) Si chips facing down and connected to the FPC. (**b**) Top view of the microfabricated 6 spin valve sensor in series, where each is 2 × 35 µm^2^. The arrow to the left of the sensor series side indicates the sensitive direction of these. (**c**) Cross-section schematic of the rigid Si chip.

The magnetic sensor elements are fabricated in large wafers using industrial processing tools at INESC-MN, individualized using a DAD 321 dicing system to their final dimensions of 0.8 × 1.5 mm^2^. After dicing, the sensors are characterized individually and selected, as a quality control measure, to secure the device performance across the eight chips in the sensing matrix. This process versatility is not available in printing or many other flexible technologies, relying on all the sensors to be viable in a matrix. The geometry for the sensors produced is shown in Figure 3.

The sensing element consists of a top-pinned spin-valve sensor microfabricated on top of a Si wafer, with the following stack (thickness in nm): Si/SiO_2_ 100/Ta 1/NiFe 2.8/CoFe 2.5/Cu 2.6/CoFe 2.3/MnIr 18/Ta 3 deposited by ion beam sputtering in a Nordiko 3000 tool [29]. Notice that both sides of the wafer are coated with the same SiO_2_ thermal oxide to minimize leakage currents through the final device’s substrate. 

To obtain a linear response from the sensor, the rectangular 2 × 35 μm^2^ spin-valve elements were defined by direct-write laser lithography (DWL2.0 Heidelberg, 405 nm wavelength diode laser) followed by ion milling (Nordiko 3600 tool, using a 0.16 A/cm^2^Ar+ beam). The metallic leads were patterned to connect 6 elements in series and therefore defining the sensor array. The metal contacts consist of 300 nm thick Al_98.6_ Si_1.0_ Cu_0.4_ film deposited by sputtering in a Nordiko 7000 tool (2 kW, 50 sccm Argon and 3.0 mTorr), capped by 20 nm Ru film in Nordiko 3600 to improve electrical contact, patterned by laser lithography and defined by lift-off. Finally, the sensor chip surface was passivated with a 100 nm Al_2_O_3_ layer deposited by magnetron sputtering, except over the contact pads, for further protection of the sensing elements of encapsulation.

### 3.3. Flexible Printed Circuit Board

A flexible printed circuit cable (FPC) was fabricated using a laminated foil with 25 µm thick polyimide and 9 µm of copper, patterned by laser lithography and wet etch. 

**Figure 4:**
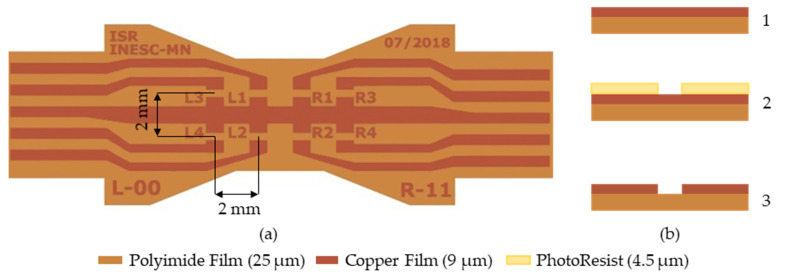
(**a**) Top view of the FPC, after defining the copper layer; (**b**) microfabrication steps: 1. Bare copper/polyimide film cleaning, 2. Coating and definition of photoresist using lithography system, 3. Wet etch and resist stripping.

**Figure 5:**
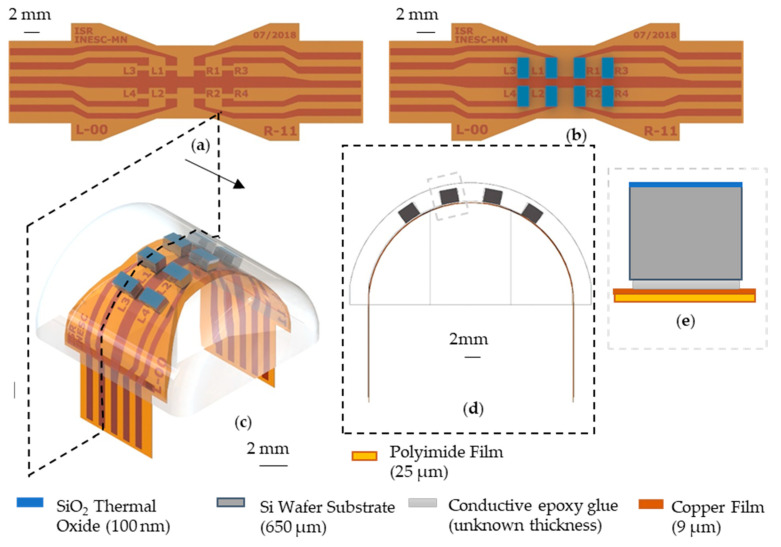
(**a**) Top view of the FPC, showing the pads to connect the Si chips; (**b**) Si chip in the FPC using the epoxy glue; (**c**) 3D view of the FPC with the Si chips embedded in the PDMS part. (**d**) Cross section of the device showing the curvature for the flexible sensing matrix. (**e**) A zoom in on a Si chip glued to the FPC.

The FPC was designed to fully cover the finger surface, the most straightforward way to achieve this is to connect the left and the right side of the finger part with a “strip”. The result is an FPC that distributes 8 sensors in a 4 × 2 matrix with 2 mm in between as presented in Figure 4. The distance is much lower than previously reported designs because we could fabricate them without packaging. Moreover, the mechanical flexibility allows us to conform the FPC to the finger surface shape resulting in mounting architecture shown in Figure 5c,d. 

The bonding of the Si chip to the FPC is an important development step for this fabrication process. The bonding must guarantee a good electrical contact and strong mechanical adhesion. For this design geometry to have a minimal footprint the Si chip must be flipped to contact the FPC (Figure 5b). Soldering Si chips facing down was not an option, and any other manual approach would have a higher footprint making it impossible to reach such high densities. Moreover, the sensor’s exposure to high temperatures (over 200 °C) will lead to signal loss from exchange bias weakening and inter-layer diffusion. The first can be reversed by cooling the sensors in a 1 T magnetic field, while the second is irreversible. Ensuring all the sensors are exposed to the same thermal cycle helps us guarantee that the signal has less influence from thermal issues. The sensor chips were flipped and glued to the FPC using a silver conductive epoxy adhesive (MG Chemicals 8331). The main challenges of this step are the manual manipulation of the relatively small Si chips and the lack of an accurate epoxy volume control. The manual manipulation can lead to two main assembly errors: the distance between the sensor to be smaller or larger than the 2 mm they were dimensioned to be; and a relative angle between them. The tested devices were assembled with a precision of ~200 µm, and angle errors under 10°. A better epoxy volume could allow us to better understand the influence of thickness and mechanical and electrical quality of the contact.

### 3.4. Polymeric Finger Part

**Figure 6:**
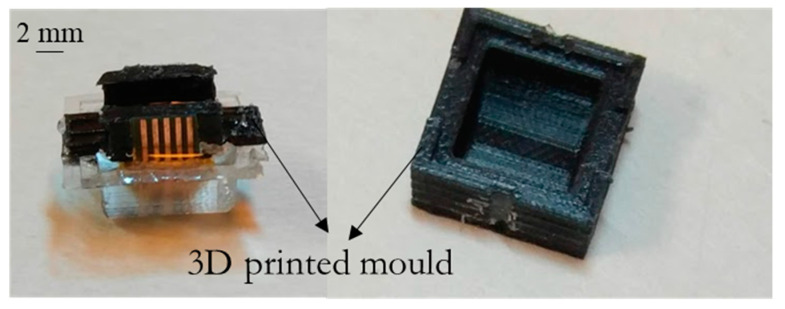
Two-part 3D printed mold used to shape the elastomeric part and conform the FPC with the Si chips.

Finally, the FPC and the sensors were embedded in an elastomer cap, shaped with similar curvature as the FPC, to protect it from the environment and provide the robot with better grasping. A Witbox 3D printer fabricated the mold using a PLA filament and a layer height of 0.2 mm. The FSM was attached to the molds, holding it in place while also serving as a casting mold for shaping the silicone cap (Figure 6). The curing of the polymer coincided with the FPC and polymer bonding. The elastomer used was PDMS (polydimethylsiloxane) in a 1:15 proportion and cured at 70 °C for 1 h. This temperature does not affect the magnetoresistive sensor, demonstrating thermal stability up to 120 °C [31].

### 3.5. Electronic Interface

**Figure 7:**
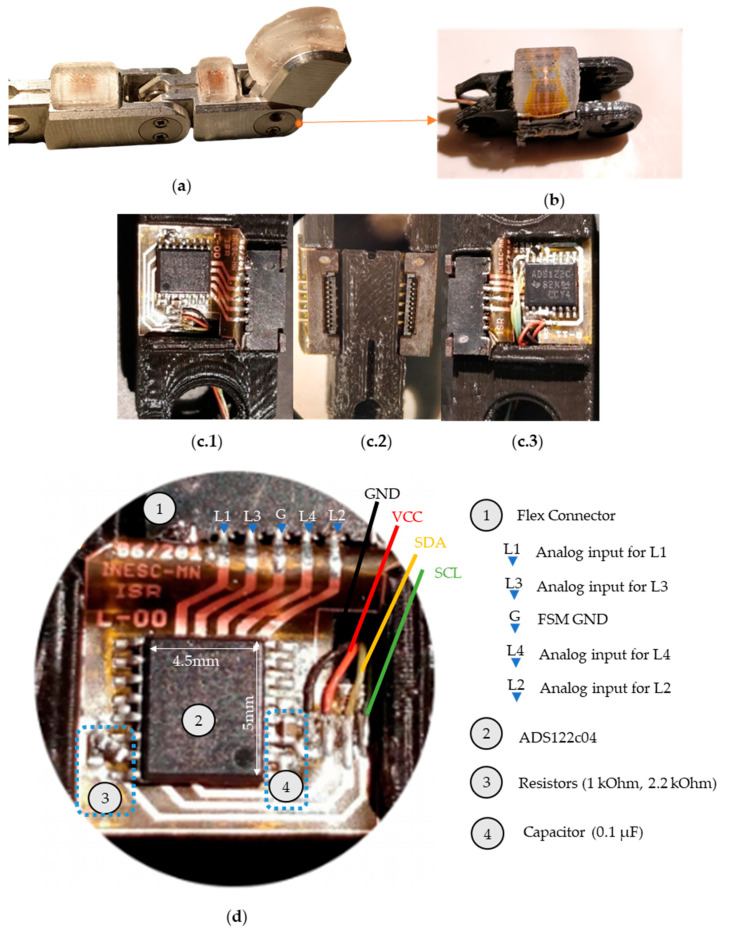
(**a**) Vizzy’s finger is made of aluminum and is compatible with Figure 1a and is described in detail in a previous work [6]. (**b**) Redesigned 3D printed prototype for the middle phalange, the electronic interface, and the tactile sensor. (**c**) The prototyped finger part without the tactile sensor, detailing the flex connectors (**c.2**); the FPC (L-00) on the left side (**c.1**) and the FPC (R-11) on the right side (**c.3**); (**d**) detail of the L-00 FPC, identifying the flex connector and the analog inputs, the ADS122c04, resistors, capacitor, and the I2C output.

The finger part chosen to test this approach was the middle phalange of Vizzy’s finger (see Figure 7a). This phalange has the lowest active area, among all phalanges, resulting in the lowest number of sensors to cover the surface and thus simpler electronics. The design and development of a custom-made solution benefits from the flexibility in design but raises some challenges regarding integration, specifically the electronic interface. 

First, we had to make sure the electronics could fit the finger part without restraining any finger, hand, or arm movements. We redesigned the aluminum part at a 1:1 scale using a PLA filament and a Witbox 3D printer (Figure 7b) to fit two extra FPCs one for each side. The left FPC (L-00) is shown in Figure 7(c.1) and the right FPC (R-11) is shown in Figure 7(c.3). These two extra FPCs are used to convert the analog signals from the flexible sensor matrix with the 8 Si chips (described in the previous section) and output into the standard I2C communication interface (GND, VCC, SDA, and SCL).

To connect the three boards to each other, we used vertical flex connectors, one for each board on the side of the finger. In order to capture the data from the sensor on each lateral FPC the main component of interest is the ADS122C04 which has a 24-bit ADC, a current source and a multiplexer. The ADS122C04 requires two 1 kΩ 0402 resistors and two 0402 100 nF capacitors, to enable the readout of 4 individual sensors with a noise peak-to-peak (Noise_P-P_) level of 25 µV, at 10 samples per second per sensor with 1 mA current. 

The digital signal output is interpreted by an Arduino MRK1000, which is responsible for communicating with the device and connecting to a computer via USB, where the data is analyzed.

## 4. Sensor Characterization

### 4.1. Si Chip

The geometrical constraints to fit the electronics in the finger limits the size of the electronic interface significantly. The ADS122c04 size (4.5 × 5 mm) occupies most of the available space itself (Figure 7), so including an amplifier was not considered possible. Without an amplifier the only choice left is to use the current to amplify the signal. The ADC has a saturation voltage of 2.048 V and can provide a maximum of 1.5 mA as current bias. Using a 1 mA current and 2 kΩ, sensor can maximize the sensor output without saturating the ADC. Therefore, we have the six spin-valve series that are tailored to have a total resistance of 2 kΩ, and a linear range of at least −1 to 1 mT. The series of six 2 × 35 µm^2^ fabricated sensors have a sensitivity of 0.72%/mT and a resistance of 2 kΩ. So, the electrical range of operation for the sensor in this application will be from 1.985 V for −1 mT to 2.015 V for 1 mT with a 1 mA applied current.

**Figure 8:**
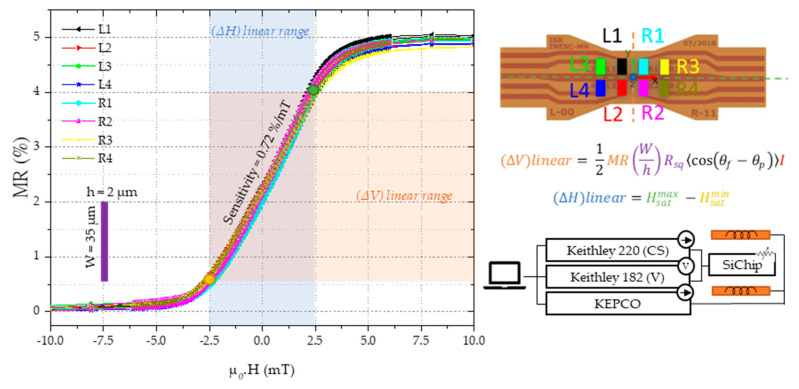
Transfer curve MR(H) curve of the used Si chips within a field range of ±10 mT with a 1 mA current. The current to the sensor was supplied by a Keithley 220 programmable current source and the voltage was measured with a Keithley 182 sensitive digital voltmeter, while the current provided to the Helmholtz coils responsible for controlling the applied field (µ_0_.H) is a KEPCO bipolar operational power supply.

The transfer curve of MR(H) of all eight sensors, L1–R4, displays a clear excellent uniformity, with an average sensitivity of 0.72%/mT (14.1 mV/mT) and the moderate magnetoresistance values MR ~5% consistent with excessive contact resistance in these series connection architectures (Figure 8). The spin-valve sensors were characterized at wafer level. The sensor bias current (1 mA) was supplied by a Keithley 220 programmable current source and the voltage measured by a Keithley 182 sensitive digital voltmeter, while a KEPCO bipolar power supply was used to set the current to the Helmholtz coils during the transfer curve MR(H) characterization. The chips used in this work show resistance in the saturation state Rmin ~2 kΩ, coercivity Hc <0.1 mT, and the transfer curves are centered around H = 0 (small shift <0.4 mT, caused by the Neel coupling and demagnetizing fields [32]) and a linear range of ±2.5 mT. 

### 4.2. Si Chip Bonding to the FPC

The use of conductive epoxy to bond the Si chips to the FPC described in Section 3.3 brought challenges such as controlling the volume of glue used per pad, the chip alignment, and the quality of the electrical contact. The quality of electrical contact can limit the detectivity. The ADC manufacturer reports a 20 µV noise peak to peak, which for this design, we could expect a 1.4 µT minimum detectable field. 

The pot life of this epoxy is about 10 min, which added an extra layer of difficulty during the Si chips’ manual placement on the FPC.

The silver conductive epoxy adhesive recommended curing instructions are: 24-h at room temperature, 15 min at 65 °C, or 7 min at 125 °C. To maximize the throughput, the 24 h curing at room temperature procedure was not considered. For the lower temperature, the curing was performed up to 60 min without significant improvement of the quality of the electric contact. However, only 10 min at 150 °C (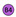) provided an electrical contact quality as good as when measuring directly on the contact pads of the chip. Lower Noise_P2P_ was achieved by increasing the temperature to 250 °C and time to 30 min (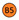), resulting in a better contact quality than provided by the probes placed on the contact pads of the Si chip. Higher temperatures and longer time make the magnetoresistive sensor prone to inter-layer diffusion and consequent loss of signal, so these are suitable process parameters for bonding.

### 4.3. Electronic Interface

**Figure 9:**
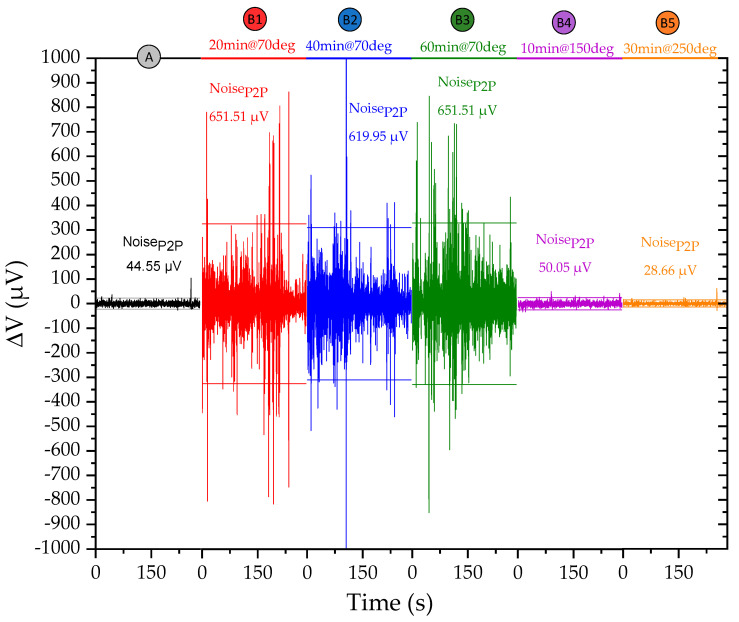
Comparison of different curing procedures. We show the voltage variation (µV) of the sensor used in L1 during a 300 s measurement at H = 0 T using a Keithley 220 as a current source, Keithley 182 voltmeter, and micrometric probes to make contact with: the contact pads of the Si chip (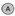), the FPC with the Si chip bonded with the epoxy cured at 70 °C for 20 min (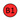), 40 min (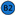) and 60 min (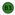), cured at 150 °C for 10 min (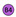) and 250 °C for 30 min (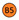).

The Si chips bonded to the FPC characterized in Figure 9, were then embedded in the PDMS as described in 3.4 and connected to the electronic interface detailed in 3.5.

**Figure 10:**
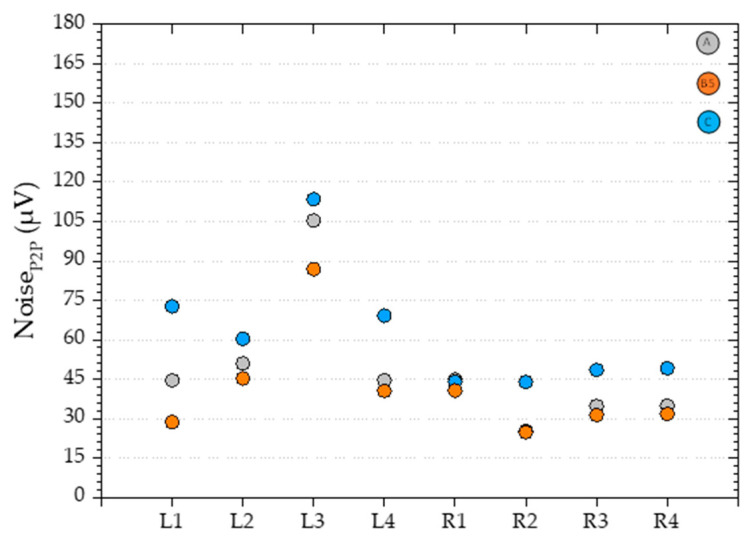
Comparison between the noise_P2P_ (µV), for all eight sensors (L1–R4), at H = 0 T using a Keithley 220 as a current source, Keithley 182 voltmeter, and micrometric probes directly on to the contact pads of the Si chip (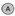), the FPC with the Si chip bonded with the epoxy cured at 250 °C for 30 min (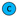) and the same but using the electronic interface described in Section 3.5 (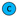).

When comparing the Noise_P2P_ before the bonding process (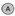) and the final device (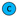), an average increase of 25% is observed across all sensors (Figure 10), which considering the benefits of integration, is considered an acceptable trade-off.

**Figure 11:**
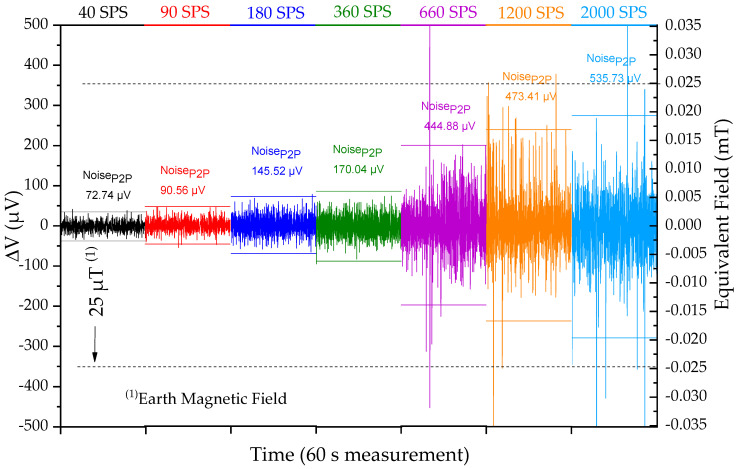
Noise_P2P_ (µV) for sensor L1 at H = 0 T in the final device (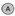) for different data rates.

The data rate (samples per second) at which one can retrieve the data from the sensor is also an important parameter. The reaction time is defined by the sum of the acquisition time, the processing time, and actuation time. Reducing the acquisition time requires higher samples per second (SPS). An increase in the noise_P2P_ is expected due to the sigma-delta ADC working principle and is characterized in Figure 11. To measure at 2000 samples per second, the detectivity changes by a factor of 8, from 5 µT to 38 µT (Figure 11). A ±25 µT reference point (dashed line) and the scale for converting to an equivalent magnetic field (mT) was added. The earth’s magnetic field can vary from ±30 to 50 µT and would still be detectable for a 2000 SPS rate. However, it limits the device’s ability to detect lower sensor displacements, and consequently, the minimum detectable force of the device.

### 4.4. Experimental Setup

**Figure 12:**
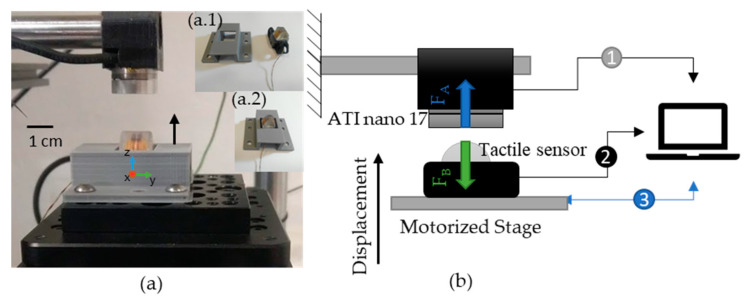
(**a**) The aluminum rod in a cantilever configuration, the ATI nano 17, the magnetic tactile sensor, and the stage; (**a.1**) shows a 3D printed part (left) to fix the tactile sensor (right) to the stage; (**a.2**) shows how the 3D printed part fits with the tactile sensor. (**b**) Force equilibrium and working principles of the setup, where the 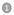 identifies data from the ATI nano 17 ($F_{x}$, $F_{y}$, $F_{z}$,$\;T_{x}$, $T_{y}\;$ and $T_{z}$), 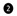 the data from both L-00 and R-11 lateral FPCS of the eight sensors (L1–R4) and 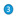 the data from the PC to the three servo motors Thorlabs stage controlling the displacement.

The transport curve and electrical characterization are essential for fabrication and manufacturing control but do not provide clear insights into the sensor operational limitations. Fully integrating the sensor on the robot hand or just the finger is a complex and time-consuming task, so it becomes valuable to test the tactile sensors as close to the application conditions as possible to an actual situation. Therefore, a setup that could apply and measure forces precisely is vital for further optimizing and developing of the tactile sensor. We assembled a setup consisting of the magnetic tactile sensor, a three-axis cartesian motorized stage (Thorlabs DDS220), and a 6-degree force sensor (ATI nano 17). The tactile sensor is fixed on the stage using a 3D printed part (Figure 12(a.1,a.2)).

The ATI nano 17 is a multi-axis force and torque sensor system that is able to measure both forces ($F_{x}$, $F_{y}$, and $F_{z}$) and torques ($T_{x}$, $T_{y},\;$ and $T_{z}$). In this setup we attached one to an aluminum cantilever fixed to the table. A computer communicates with the stage, providing instructions to align and press the sensors against each other while reading their output. This setup configuration allows an evaluation of the sensor performance under normal and shear forces. For this case, the tactile sensor curved surface top-center point was aligned with the ATI nano sensor, and the stage presses the sensors against each other.

**Figure 13:**
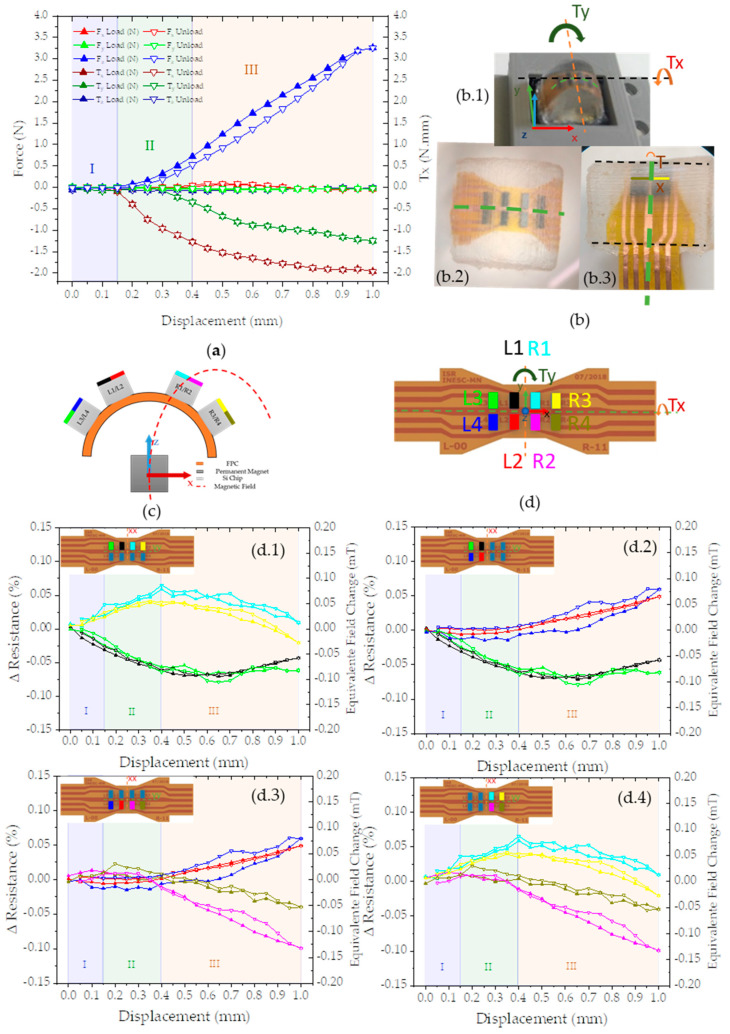
(**a**) Force and momentum data from ATI nano 17 ($F_{x}$, $F_{y}$, $F_{z}$,$\;T_{x}$, $T_{y}\;$ and $T_{z}$) for the loading and unloading phases. (**b**) Detail of the sensor with the $T_{x}$ and $T_{y}$ schematics detailed. (**c**) A schematic view of the sensor matrix in the XZ plane; (**d**) a schematic view of the sensor matrix in the XY plane, showing the sensors; (**d.1**) set of four graphs showing the symmetry for the sensor output by choosing sensors: (**d.2**) with positive y coordinates (odd numbers—L1, R1, L3, R3); (**d.2**) with negative x coordinates (left side—L1, L2, L3, L4) (**d.3**) with negative y coordinates (even numbers—L2, R2, L4, R4); (**d.4**) with positive x coordinates (right side—R1, R2, R3, R4).

The sensors are aligned visually in such a way that the tactile sensor touches the ATI nano center. The alignment results from actuating the stage and visually checking whether they are centered in X and Y directions and touching in the Z direction. After alignment, the stage moves vertically in steps of 0.05 mm up to 1 mm, pressing the sensors against each other. The recorded values from the ATI nano and tactile sensor for each step are plotted in Figure 13a,b, respectively. The load phase corresponding to pressing the tactile and force sensors against each other is followed by an unload phase, where the opposite occurs.

The stage position changes every 30 s providing the time frame to record the sensor’s output. The values presented in Figure 13(d.1–d.4) show the average value for each position. The ATI nano output already considers a calibration from the manufacturer to provide the force and torque output in Figure 13a. The tactile sensor output comprises individual voltage measurements of the eight Si chips. We use the percentage of change in resistance, ΔResistance (%), to quantify the applied magnetic field change (equivalent field change (µT)).

The percentage of change in resistance is the arithmetic average for a sensor when the stage is in a position (p) relative to the arithmetic mean of the same sensor for the initial position of the stage, without any applied force.

(1) $$\Delta R_{p}\left(\%\right)=\frac{\bar{R_{pi}}-\bar{R_{p=0,\;i}}}{\bar{R_{p=0}}}\times 100\;$$

(2) $$\mathrm{Equivalent}\;\mathrm{Field}\;\mathrm{Change}\;\left(\mathrm{mT}\right)=\frac{\Delta R_{p}\left(\%\right)}{Sensitivity\;\left(\frac{\%}{mT}\right)}$$

The equivalent field change represents the change in magnetic field aligned with the sensor plane and perpendicular to the pinned layer and we calculate it using Equation (4). The change in resistance results from Equation (3), while the value used for sensitivity is 0.72%/mT. The value of sensitivity is the average of the individual sensor sensitivity obtained from the slope of the MR(H) curve shown in Figure 8. The Si chips have a spin-valve sensor series sensitive to magnetic fields in the x-axis and the permanent magnet aligned with the FPC center. This configuration results in the sensor output of Figure 13(d.1–d.4), where sensors on the left side detect a decrease in a magnetic field (d.2) while the sensors on the right side show the opposite behavior (d.4).

The values recorded can be divided into three main phases: an initial adjustment phase (I—blue), a torque-dominated phase (II—green), and a vertical force-dominated phase (III—orange). The initial adjustment phase (I—blue) occurs in the first 150 µm of vertical stage displacement. In this phase, the forces are below the detection limit of ATI nano 17 but detectable by the tactile sensor since the tactile sensor matrix measures a change in a magnetic field coherent with the displacement, both for loading and unloading. Moreover, the sensor output has a precise symmetry, with positive y coordinates (odd numbers—L1, R1, L3, R3) and negative y coordinates (even numbers—L2, R2, L4, R4). We attribute this phase to gaps present in the whole experimental setup system that require less force than the minimum force the ATI nano can detect.

The second phase (II—green) occurs from 0.15 to 0.4 mm, and we observe a more significant increase in $T_{x}$ than in $F_{z}$, thus we have named it a torque-dominated phase. Torque was not intentionally imposed, but it is consistent with a misalignment during production or assemblage of the sensor matrix (see Figure 13(b.3)). The tactile sensor’s output is also consistent with a rotation in the x-axis, which can be observed by comparing the outputs in Figure 13 d.1 with d.3. In phase II the sensors with positive y-coordinates (odd numbers—L1, R1, L3, R3) show a positive change, while negative y-coordinates (even numbers—L2, R2, L4, R4) show no observable output.

The last phase (III—orange) where $F_{z}$ becomes a more significant factor than any other force or torque, from 0.4 to 1 mm, $F_{z}$ has a similar behavior to the simulated force (Figure 15f).

### 4.5. Simulating the Experimental Setup

#### 4.5.1. Simulation Assumptions

**Figure 14:**
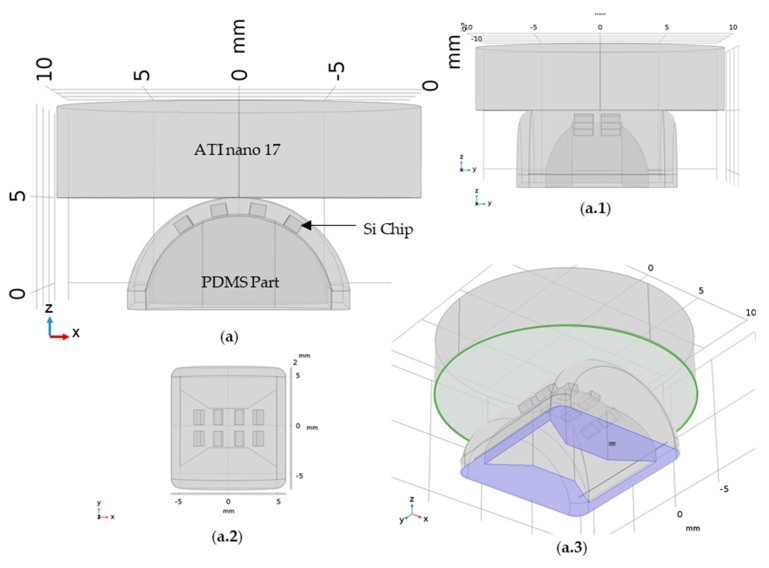
Three-dimensional geometry used for the simulations: (**a**) XZ plane view; (**a.1**) YZ plane view; (**a.2**) XY plane view; and (**a.3**) an orthogonal view identifying the initial constraints, the blue represents the finger part on the Thorlabs stage and thus was fixed, while the green area highlights the surface that will be pressed against the PDMS part in steps of 0.05 mm up to a maximum of 0.5 mm.

To improve our understanding of the results and accelerate the development and optimization of new designs, we represented and simulated the same system in three dimensions using finite element software, COMSOL. The model only considers the Si chips, the elastomeric part, and the ATI nano 17 (Figure 14). The FPC, the epoxy glue, and the finger part are not considered in the model since they would significantly increase the computational complexity and their contributions are most likely negligible. The main purpose of this simulation is to explore the relationship between the displacement and the magnetic field detected in the surface of the Si chips (related to the sensor output in the experimental data). This relationship represents the working principle of the device, thus, understanding the metrics that have the most impact on the working principle allows us to develop better-suited devices for the application. Moreover, simulation decreases the iteration time significantly, thus connecting the experimental data and simulation data is key for an efficient design and development of these sensors.

The simulation comprises: (i) a mechanical model, where the bottom of the elastomeric part was fixed (blue area in Figure 14(a.3)) and the ATI nano 17 was pushed against it in steps of 0.05 mm until 0.5 mm and (ii) a magnetic model, where the magnetic field H (Hx, Hy, Hz) was calculated for each Si chip. 

#### 4.5.2. Mechanical Simulation

The mechanical model simulates the deformation of the PDMS part when pressed by the ATI nano 17, and consequently how the Si chips are displaced. 

The most challenging aspect of this simulation was defining the interface behavior correctly. The interface between the ATI nano and the elastomeric part had to be defined in such a way that allowed for the area of contact to change as the ATI nano displacement increased. This was achieved by using the augmented Lagrange pressure method available in COMSOL 3.5 to simulate the evolution of the contact area. To simulate the deformations of the PDMS part, an incompressible neo-Hookean hyperelastic material model was used in COMSOL [33]. The parameters used for the mechanical simulations are detailed in Table 1.

**Figure 15:**
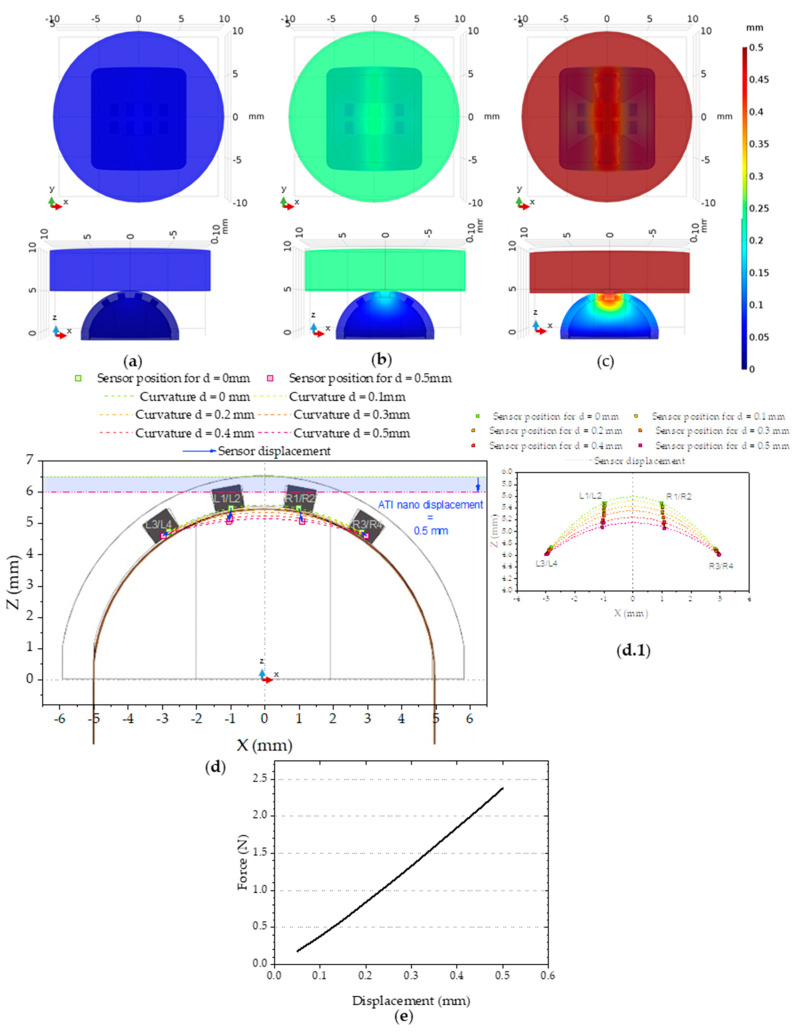
Results of the three-dimensional mechanical simulation detailing the XY plane and ZX plane and showing the displacement results for the steps in color: (**a**) 0.05 mm; (**b**) 0.25 mm; and (**c**) 0.5 mm. (**d**) XZ plane showing the position and displacement of the Si chips. (**d.1**) Detail of the matrix displacement for each pressing step. (**e**) The force value, obtained by integrating the value of force in each point of the green surface on Figure 14(a.3).

In this simulation we forced a vertical and controlled displacement of the aluminum cylinder to compress the elastomeric part. This displacement forced the deformation of the elastomeric part and a reaction force on the aluminum part. The total force measured in the green surface is plotted against the displacement on Figure 15e can be compared with the experimental data in 13a. The mechanical model seems to describe the experimental data acceptably, particularly if we consider the phase I 150 µm gap discussed in the experimental data. The simulation was only done for 0.5 mm to make sure we were working in the elastic domain so the model could have any significance.

#### 4.5.3. Magnetic Simulation

The magnetic simulation is performed for each displacement step because for each displacement a different position in space results in a different magnetic field. The magnitude of the magnetic field generated by the cylindrical NdFeB magnet is simulated and measured on the active area of the Si chips for each displacement. 

**Figure 16:**
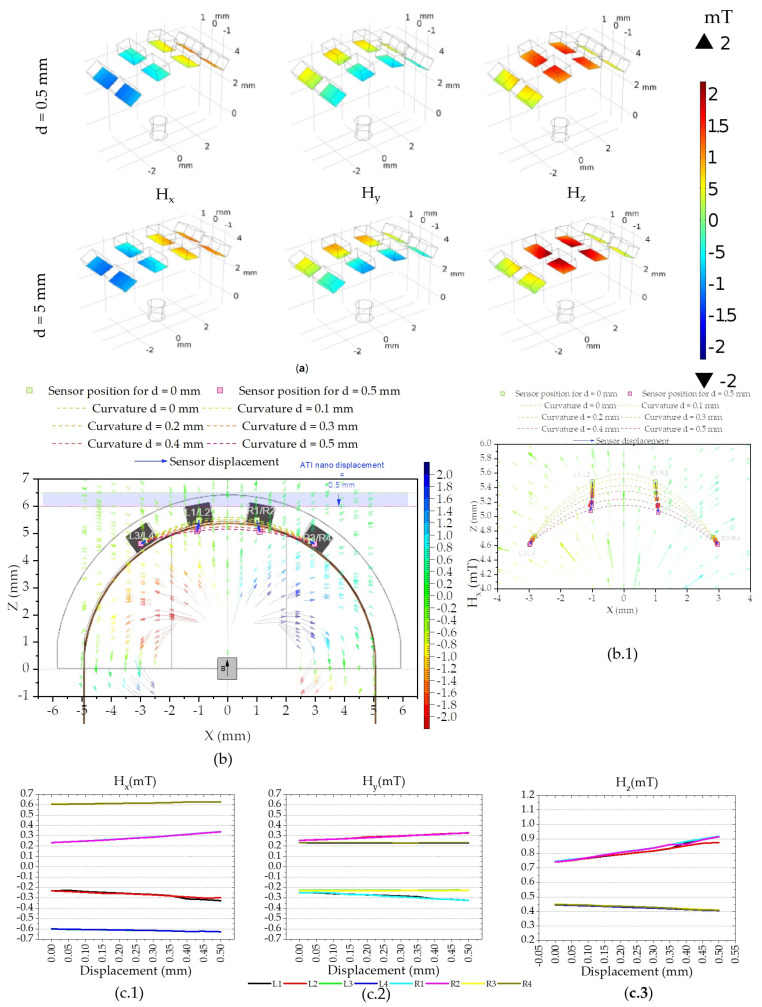
Three-dimensional magnetic simulation, showing (**a**) the Si chip and the field magnitude in Hx, Hy, and Hz magnitudes on the surface where the sensors for d = 0 and 0.5 mm. (**b**) XZ plane showing the position and displacement of the Si chips, the position of the magnet, and the magnetic field lines. (**b.1**) Detail of the matrix displacement for each pressing step. (**c**) Average magnetic field magnitude calculation in each Si chip in their active sensor area (200 × 95 µm^2^) for each component in µT: (**c.1**) $H_{x}$ (**c.2**) $H_{y}$ and (**c.3**) $H_{z}$.

For the geometrical configuration of the tactile sensor the magnetic field ranges between −2 and 2 mT on the surface of the Si chips where the sensor is fabricated (colored surfaces in Figure 16a). 

The transfer curve (Figure 8) shows that the sensors respond linearly to magnetic fields ranging from −2.5 to 2.5 mT perpendicular to their easy axis. However, each point of the colored surfaces (Figure 16a) is a three-dimensional vector of magnetic field with origin at the permanent magnet. To calculate the field detected by the sensor, and be able to compare the simulation to the experimental data, we must consider the active area of the sensors and the angle of the surface with respect to the XY plane. 

The active sensor area of 200 × 95 µm^2^ (see in Figure 4b) in the center of the Si chip is used for determining the magnetic field in further calculations such as Figure 16c, as opposed to the average of the colored surface as a whole. In the same figure we can see a linear relationship between the magnetic field and the displacement. Moreover, symmetry between the position of the sensors in the matrix and the simulated magnetic fields is expected, as sensors on the left side (negative x-coordinates) show a negative Hx and sensors with a positive y-coordinate show a positive Hy.

#### 4.5.4. Sensor Tilting

In addition to the sensor’s active area position in space we must consider the rotation angle of the Si chip surface with respect to the permanent magnet referential in three dimensions (α,β, γ). The Si chip surface plane with the XY plane is referred as α. The green referential frame represents the magnetic simulation system of coordinates (x,y,z), with origin at the center of the magnet. The blue referential (a,b,c) has its origin in the center of the sensor and is defined with $\;{\vec{e}}_{a}$ and ${\vec{e}}_{b}$ being orthogonal and lying in the sensor plane and ${\vec{e}}_{a}$ aligned with the sensor sensitive direction. Because the sensor is only sensitive in one direction on the plane and the field is a vector in the three dimensions, taking the angle (α,β, γ) of the Si chip with the permanent magnet is needed to evaluate the magnitude of the sensor output, henceforth ${\vec{H}}_{sensor}$. To determine its value, we use the following:

(3) $${\vec{H}}_{sensor}=R_{x}\left(\alpha \right)\;R_{y}\left(\beta \right)\;R_{z}\left(\gamma \right)\;\vec{H}\;$$

(4) $$\left[\begin{array}{c} H_{a}\\ H_{b}\\ H_{c} \end{array}\right]=\left[\begin{array}{ccc} 1 & 0 & 0\\ 0 & \cos\alpha & -\sin\alpha \\ 0 & \sin\alpha & \cos\alpha \end{array}\right]\;\left[\begin{array}{ccc} \cos\beta & 0 & \sin\beta \\ 0 & 1 & 0\\ -\sin\beta & 0 & \cos\beta \end{array}\right]\;\left[\begin{array}{ccc} \cos\gamma & -\sin\gamma & 0\\ \sin\gamma & \cos\gamma & 0\\ 0 & 0 & 1 \end{array}\right]\;\left[\begin{array}{c} H_{x}\\ H_{y}\\ H_{z} \end{array}\right]$$

**Figure 17:**
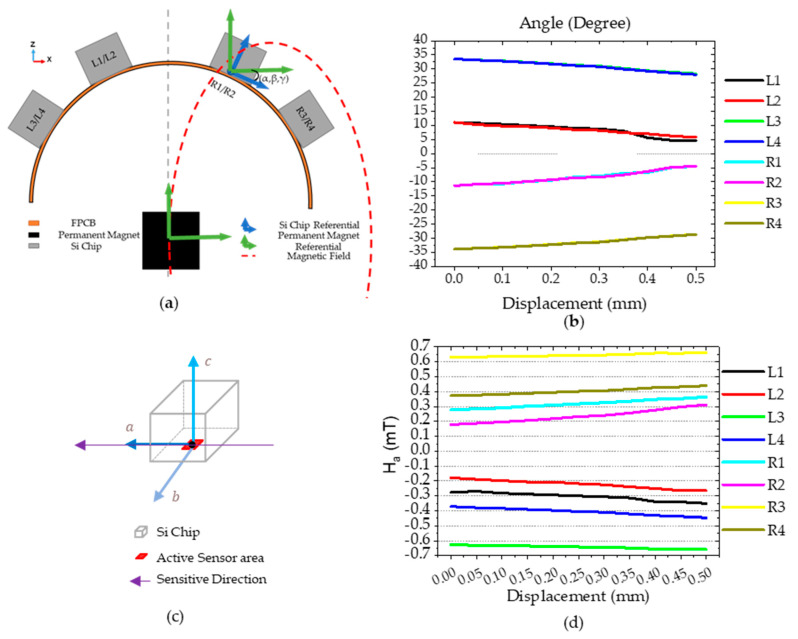
(**a**) XZ plane schematics showing the relationship between the Si chip (blue) and permanent magnet (green) reference frame; (**b**) angle α simulated and taken from the mechanical simulation for each Si chip, in this case both β and γ are 0. (**c**) abc referential centered on the spin valve active area, which coincides with the Si chip’s surface. (**d**) The magnetic field magnitude on the active area of the sensor.

The α values obtained in the simulation for each Si chip are shown in Figure 17b. For this case, the angles β and γ  are considered null as no change was observed in this simulation. This is attributed mainly to the pressing object flat geometry that has an area larger than the sensor as well as the applied force only has a vertical component. Smaller or curvilinear pressing objects would have a significant impact on β, while either torques, shear forces, or both, would impact γ. 

To estimate the ${\vec{H}}_{sensor}\;\left(H_{a},H_{b},H_{c}\right)$ we use Equation (3), where $\vec{H}\;\left(H_{x},H_{y},H_{z}\right)$ is the magnetic field vector for the coordinates in 3D space corresponding to the center of the sensor’s active area. The matrices in (4) are used to obtain $\vec{H}$ in the abc referential. 

The ratio between $H_{a}$ and $H_{b}$ is 2:1 and the fact that $H_{b}$ << 1 mT, suggest that $H_{b}$ influence due to crossfield phenomena on the sensor output can be discarded. The values of $H_{a}$ against the displacement are shown in Figure 17c. With these calculations we estimate $H_{a}$ range between −0.7 and 0.7 mT, which is well within the linear range of the sensor-transfer curves presented in Figure 8. 

### 4.6. Simulation and Experimental Data, How Do They Compare?

Section 4.4 discusses the three phases of the physical experiment and how the change in resistance value is obtained. This means that a change of 0.05% in the signal of the sensor should correspond to a 50 µT change. In order to compare this with the simulation results, we need: (1) only use the data from phase III of experimental data; and (2) to use the same principles for the simulation data, meaning we need to look at changes in magnetic field.

**Figure 18:**
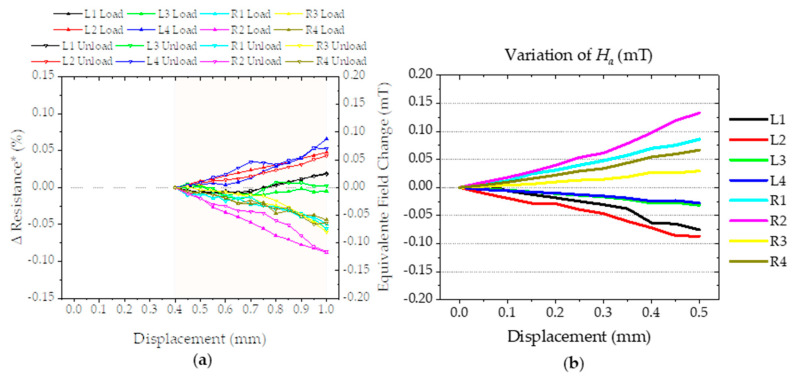
(**a**) Experimental results: sensor output as a function of displacement for phase III. (**b**) Simulated results considering change in angle.

The first part implies that we plot the change of resistance discarding phases I and II and assume the $\bar{R_{p=0}}\;$ in Equation (3) to be p=0.4 instead of p=0 (Figure 18a). As for the simulated data, we use $H_{a}$ and the field for the position to be clearly visualize the similarities between both sets of data; Figure 18b shows the variation of the $H_{a}$ as a function of displacement for each sensor. This can be compared directly with the equivalent field change (µT). The simulation seems to predict the experiment accurately for the sensors L2, R1, R2, and R4. 

## 5. Conclusions

We presented an innovative design for a tactile sensor integrated into the fingertip of a robotic hand, based on magnetic sensing, and leveraging a working principle that was not attempted in the literature so far. The main advantage of this working principle is the ability to pin-point the exact source of displacement by measuring the displacement of the sensors on the surface of the finger. This was possible thanks to a comprehensive simulation of the behavior of the different components of the sensor, that allowed optimizing of the design of the system and to obtain the desired performance, and an effective miniaturization of the physical components.

The influence of external fields is a concern for this application. We believe there are two main strategies to mitigate errors from this source. The first is to add a magnetic shield between the sensors and the external field, which would mitigate the influence of external magnetic fields. Magnetic shields can be made from materials with high permeability that can be used to manipulate magnetic field lines either of magnetic flux concentrators to amplify small magnetic signals [34], or the opposite. Mu metal (nickel-iron alloy) is a common material used in magnetic shielded cameras for low noise measurement.

The other is to use algorithms to treat the information of the sensors and this can determine the probability that the change in magnetic field was caused by an external source or due to a deformation of the elastomer. Knowing the characteristics of the cylindrical permanent magnet and expected position of each sensor, allows us to have a reasonable expectation regarding the flexible sensing response. In cases where this does not match, it is highly likely that an external magnetic field is present. In a situation where all the sensors detect the same change in magnetic field (i.e., earth magnetic field) would result in an offset, for all the sensors simultaneously. A constant offset in the same magnitude and direction for all the sensors is not the expected behavior when a deformation occurs (as discussed in Section 4.4). The symmetry of the magnetic field from the cylindrical permanent would allow us to filter it. An example where such a strategy is successfully used is when minimizing magnetic noise for magnetoencephalography (MEG) equipment, where advanced mathematical algorithms applied on the multichannel MEG data are used to minimize external field interference [35].

The number of sensors is a critical parameter for this design. There are two reasons for increasing the number of sensors: improved ability to identify sources of external fields and better ability to detect the contact point.

## Figures and Tables

**Table 1:** Parameters used for each material in the mechanical simulation.

Mechanical Properties			
Part	PDMS	ATI Nano 17	Si Chip
Material	PDMS—Polydimethylsiloxane (1:15)	Aluminum	Silicon (solid, [100] axis)
E	750 kPa	6.91 GPa	13.02 GPa
u	0.49	0.33	0.28
K	-	25.98 GPa	79.67 GPa
µ	251.68 N/mm^2^	-	-
l	12.33 kN/mm^2^	-	-

E = Young modulus; υ = Poisson ratio; K = Bulk modulus; Lamé parameters—µ and λ.
